# A Case of Chronic Ethylene Glycol Intoxication Presenting without Classic Metabolic Derangements

**DOI:** 10.1155/2014/128145

**Published:** 2014-08-21

**Authors:** Stephanie M. Toth-Manikowski, Hanni Menn-Josephy, Jasvinder Bhatia

**Affiliations:** ^1^Department of Medicine, Boston Medical Center, 72 East Concord Street, Evans 124, Boston, MA 02118, USA; ^2^Renal Section, Boston University School of Medicine, 650 Albany Street, Rm 504, Boston, MA 02118, USA

## Abstract

Acute ethylene glycol ingestion classically presents with high anion gap acidosis, elevated osmolar gap, altered mental status, and acute renal failure. However, chronic ingestion of ethylene glycol is a challenging diagnosis that can present as acute kidney injury with subtle physical findings and without the classic metabolic derangements. We present a case of chronic ethylene glycol ingestion in a patient who presented with acute kidney injury and repeated denials of an exposure history. Kidney biopsy was critical to the elucidation of the cause of his worsening renal function.

## 1. Introduction

Ethylene glycol is a colorless, odorless, sweet-tasting chemical found in products such as automotive antifreeze, windshield wiper fluid, solvents, cleaners, and other industrial products. Ingestion can lead to central nervous system depression, organ dysfunction, and ultimately death if left untreated [1].

In the acute setting of ethylene glycol ingestion, diagnosis in an intoxicated individual is confirmed by profound anion gap metabolic acidosis, elevated osmolar gap, and elevated ethylene glycol level. However, chronic ingestion of small amounts of ethylene glycol presents a diagnostic conundrum because laboratory derangements are often absent and physical symptoms can be mild. To date, very few case reports describe chronic ethylene glycol ingestion.

A review of the literature demonstrates common themes in chronic ethylene glycol ingestion. Patients present with acute kidney injury and a mildly elevated anion gap that resolve with minimal intervention [2, 3]. They describe abdominal discomfort that ranges from nausea, vomiting, and diarrhea to abdominal cramping [1, 3–5]. Cases are also notable for a prior history of substance abuse or a history of depression [1–3, 6].

We present a case of chronic ethylene glycol ingestion in a patient who presented with unexplained acute kidney injury, abdominal complaints, and anion gap acidosis which resolved quickly with supportive therapy. Ultimately, kidney biopsy was essential in revealing the etiology of his worsening renal function. This case illustrates the need for a high index of suspicion of intoxication despite the lack of history, minimal symptoms, and lack of classic lab abnormalities.

## 2. Case Presentation

A 41-year-old man with a history of hypertension, migraines, stroke, and depression presented to the emergency department complaining of five days of abdominal pain, nausea, and vomiting. Initial workup in the emergency room revealed a creatinine elevation of 696 *μ*mol/L (7.9 mg/dL) from a baseline of 80 *μ*mol/L (0.9 mg/dL) six weeks before. His anion gap was elevated to 19, but otherwise there were no electrolyte abnormalities. His ABG were indicative of combined metabolic acidosis and mild respiratory acidosis. The pH was 7.28, with HCO^3^ of 14.7 mmol/L (mEq/L) and PCO^2^ of 32 mmHg. The patient was not hypoxic at that time. The Ca level of the patient on the day of his admission was 9.2 mg/dL, with ionized calcium of 4.6 mg/dL. During his hospital stay, his calcium level was checked on a daily basis and was always at the normal range. His physical exam was unremarkable and his vital signs were stable. He reported taking atenolol for hypertension, topiramate for migraines, and clopidogrel and simvastatin for his history of stroke. He denied any tobacco, alcohol, or illicit drug use and he denied a family history of renal disease.

On admission, the patient was oliguric and was given normal saline at 200 mL/hour. Within 24 hours, his urine output had increased to >100 cc/hour and anion gap had normalized. Despite these improvements, his creatinine continued to rise, peaking at 1,370.2 *μ*mol/L (15.5 mg/dL). Urine sediment showed nonpigmented granular casts without cellular casts or crystals. A renal ultrasound revealed normal sized kidneys without hydronephrosis or nephrolithiasis. A renal magnetic resonance angiogram was notable for loss of renal corticomedullary differentiation but was otherwise unremarkable. Complement levels were normal, and serologies for ANA, anti-GBM, ANCA, HIV, and hepatitis A, B, and C were negative. Serum and urine immunofixation were also unremarkable.

During his hospitalization, the patient continued to complain of abdominal pain. A computed tomography scan of his abdomen was performed but did not demonstrate an intra-abdominal process to explain his persistent pain. In an effort to further work up his acute kidney injury, the patient underwent kidney biopsy.

**Figure 1:**
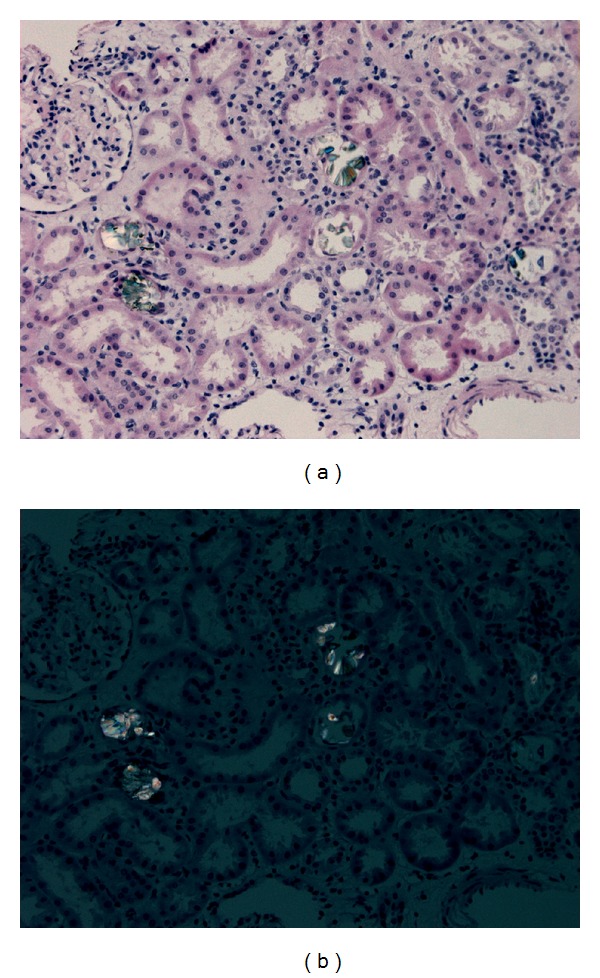
(a) Tubules packed with oxalate crystals. H&E staining, 20x magnification. (b) Birefringent oxalate crystals. H&E staining, 20x magnification, using polarized light.

A core sample submitted for microscopy revealed multiple tubules packed with birefringent oxalate crystals (Figures 1(a) and 1(b)). The tubules revealed degenerative changes of the epithelium, characterized by distention of the lumen, a low cuboidal epithelial lining, and vacuolization of the cytoplasm. The interstitium showed mild focal inflammation, with infiltrates composed primarily of mononuclear elements. There were no signs of active glomerulitis and the basement membranes appeared normal in thickness. On immunofluorescence, there was no glomerular or tubulointerstitial staining for immunoglobulins A (IgA), G (IgG), and M (IgM), C1q, C3, albumin, fibrin-related antigens, or *κ* and *λ* light chains. On electron microscopy, rare irregular electron densities are observed in the subendothelium in isolated capillaries, thought to represent entrapment of macromolecules rather than immune complexes.

A diagnosis of extensive oxalate crystal deposition in the tubules was made with associated signs of acute tubular injury and mild focal interstitial inflammation indicative of a hyperoxaluric state.

The patient was confronted about ingestion of ethylene glycol but he adamantly denied any intentional ingestions. With supportive care, the patient's kidney function gradually improved and the metabolic acidosis resolved. Upon discharge, the patient was no longer oliguric and had a creatinine of 380 *μ*mol/L (4.3 mg/dL).

He was seen one week later in renal clinic and reported persistent abdominal discomfort and nausea since discharge. The issue of ethylene glycol was once again raised, but the patient again denied any intentional toxic ingestions. A renal follow-up appointment, one month after his hospital admission, revealed a normalized creatinine of 106 *μ*mol/L (1.2 mg/dL) and normal anion and osmolar gaps. An outpatient esophagogastroduodenoscopy for the persistent abdominal pain did not reveal a source for his continued discomfort.

Seven months after his initial admission to the hospital, the patient was brought to the emergency room after being found unresponsive in a hotel room. He had recently ingested ethylene glycol and various pills in an attempt to end his life. His mental status was altered. Laboratory values revealed HCO^3^ of 5 mmol/L (mEq/L) and creatinine of 159 *μ*mol/L (1.8 mg/dL). He had an anion gap of 25 and an osmolar gap of 45. Urine sediment revealed calcium oxalate crystals, and his ethylene glycol level was 94 mg/dL (15.2 mmol/L). The patient was intubated. Emergent hemodialysis and fomepizole were started for ethylene glycol toxicity. His creatinine peaked at 689.5 *μ*mol/L (7.8 mg/dL). Upon extubation, he reported chronic ingestion of small amounts of ethylene glycol over the past several months, including the time period prior to his initial hospital admission. His creatinine on hospital discharge was 353.6 *μ*mol/L (4 mg/dL). He was discharged to a psychiatric facility and, unfortunately, was lost to outpatient followup.

## 3. Discussion

In the United States, 5,400 ethylene glycol exposures were reported to poison control centers in 2005; 700 of them were intentional ingestions [7]. Like other alcohols, ethylene glycol is rapidly and completely absorbed after oral ingestion and reaches peak serum concentrations within one to two hours [8]. Within 12–24 hours, it is metabolized to its toxic metabolites, glycolic acid, glyoxylic acid, and oxalate. At this point, an anion gap remains because of the presence of the metabolites, but the osmolar gap has resolved [9, 10].

Therefore, a delay in presentation may lead to the absence of an osmolar gap. Similarly, ingestion of small amounts of ethylene glycol may present with only a mild anion gap. Together, these observations can lead a clinician away from suspecting ethylene glycol ingestion.

Whereas ethylene glycol poisoning is a well-known clinical entity characterized by neurologic, pulmonary, and cardiovascular symptoms, chronic ethylene glycol ingestion is less common and does not have classic clinical findings [10]. In hindsight, our patient's admitting symptoms were related to chronic ethylene glycol ingestion, a diagnosis that became evident only after the kidney biopsy was performed and his subsequent suicide attempt. In reviewing the literature, we have noted a few common presenting themes that may aid the clinician in making a presumptive diagnosis of chronic ethylene glycol ingestion when laboratory findings are inconclusive and biopsy is not immediately indicated.

Patients generally describe abdominal discomfort that ranges from nausea, vomiting, and diarrhea to abdominal cramping [1, 3–6, 11]. In addition, medical history may be notable for substance abuse or mood disorders [1–3, 6]. Initial laboratory results may be notable for acute kidney injury and anion gap metabolic acidosis that resolves with minimal medical intervention [1–3]. An osmolar gap may not always be present especially if ethylene glycol ingestion is minimal and presentation to a healthcare setting is delayed [1, 11, 12]. Urine analysis may also be unremarkable [1–3].

In summary, diagnosing chronic ethylene glycol ingestion is not straightforward. Our patient presented with vague abdominal complaints, acute kidney injury, and anion gap metabolic acidosis which improved quickly and were attributed to volume depletion and acute kidney injury. Ultimately, a renal biopsy revealing oxalate crystal deposition was critical to making the diagnosis. Despite the biopsy results, our patient continued to deny intentional ingestion of ethylene glycol and only after his suicide attempt did he admit repeatedly ingesting small amounts. This case underscores the importance of keeping a high level of clinical suspicion for chronic ethylene glycol ingestion despite a lack of exposure history and significant laboratory gaps, particularly in high risk patients with a history of depression, abdominal pain, and unexplained acute kidney injury.
